# Image-based promoter prediction: a promoter prediction method based on evolutionarily generated patterns

**DOI:** 10.1038/s41598-018-36308-0

**Published:** 2018-12-06

**Authors:** Sheng Wang, Xuesong Cheng, Yajun Li, Min Wu, Yuhua Zhao

**Affiliations:** College of Life Sciences, Zhejiang University, Hangzhou, Zhejiang ZJ310058 China

## Abstract

Prediction of promoter regions is crucial for studying gene function and regulation. The well-accepted position weight matrix method for this purpose relies on predefined motifs, which would hinder application across different species. Here, we introduce image-based promoter prediction (IBPP) as a method that creates an “image” from training promoter sequences using an evolutionary approach and predicts promoters by matching with the “image”. We used *Escherichia coli* σ70 promoter sequences to test the performance of IBPP and the combination of IBPP and a support vector machine algorithm (IBPP-SVM). The “images” generated with IBPP could effectively distinguish promoter from non-promoter sequences. Compared with IBPP, IBPP-SVM showed a substantial improvement in sensitivity. Furthermore, both methods showed good performance for sequences of up to 2,000 nt in length. The performances of IBPP and IBPP-SVM were largely affected by the threshold and dimension of vectors, respectively. The source code and documentation are freely available at https://github.com/hahatcdg/IBPP.

## Introduction

Promoters are the most crucial elements in the process of transcription initiation and regulation in prokaryotes and eukaryotes. In bacteria, RNA polymerase (RNAP) and its associated sigma factors have to recognize and bind to certain regions in promoters in order to initiate transcription. The binding of RNAP and promoters is tightly regulated in bacteria and is the key mechanism modulating gene expression^1^. Therefore, accurate annotation of promoter regions in the genome is essential for studying the regulation and expression of bacterial genes. Moreover, in bacteria, functionally related genes are usually clustered into a single transcriptional unit called an operon^2^. Therefore, recognition of promoters can also facilitate the identification of operons, which would be useful for discovering the functions of unknown genes.

The position weight matrix (PWM) method is the most well-known prediction tool for identifying consensus elements in a promoter sequence^3^. Such elements include the −10 and −35 hexamers as well as binding sites for transcriptional regulators surrounding the core promoter. In addition to the sequence information for these elements, the distance between them is another important indicator for identifying promoters. Because the PWM method is limited by high false-positive rates, other computational methods have been applied for motif recognition, including hidden Markov models (HMMs)^4^. As more and more transcription factor-binding sites are discovered, the precision of these motif-based promoter prediction methods has greatly improved.

Theoretically, by fully illustrating and mimicking the mechanism through which bacteria recognize promoters, it would be possible for feature-based classification algorithms to perfectly predict promoters. However, despite great progress in our understanding of bacterial transcription initiation, the complete mechanism remains to be elucidated. Moreover, because almost all of our current knowledge on bacterial promoter features has been obtained from investigation of only a few species, such as *Escherichia coli*, there is no guarantee that the same prediction performance will be achieved for different bacterial species using these motifs. To overcome this limitation, promoter predictors that do not rely on predefined motifs are needed.

Machine-learning methods can draw information from experimentally characterized transcription start sites (TSSs), such as data derived using RNA-Seq, which can then be applied to uncharacterized sequences. In the last two decades, several machine-learning methods have been applied to prokaryote promoter prediction, including support vector machine (SVM) models^5–7^, artificial neural networks (ANNs)^8,9^, and HMMs^10^. For example, the ANN-based program NNPP2.2^11^ has been trained on both prokaryote and eukaryote promoters. Other examples of ANN-based programs include BacPP^8^, which was trained for *E. coli* promoters, and Dragon Promoter Finder^12^, which was trained on vertebrate promoters. SVM is as popular as ANNs in promoter prediction. Many researchers, including Gordon *et al*.^6^, Jiang *et al*.^13^, and Towsey *et al*.^5^, have applied SVM to the prediction of both prokaryotic and eukaryotic promoters. To apply ANNs or SVM, promoter regions are usually transformed into numerical vectors. In some cases, additional features, such as the distance between the translation start site and transcription start site, are added to the input vectors^2^. Other machine-learning methods, such as genetic programming^14^, and naive Bayes classifiers^15^, have also been applied to promoter prediction. In addition to being used alone, the abovementioned machine-learning methods can also be used in a hybrid manner. In the work of Mann *et al*., a combination of HMM and ANNs was used to identify prokaryote promoters^16^.

In addition, a deep-learning method has been used for promoter prediction, resulting in a dramatic improvement in recognition accuracy^17^. Machine-learning methods are powerful for classifying different types of data. Instead of alphabetized data, machines tend to accept digital vectors as input. Among the numerous trials of machine learning in promoter prediction, there is no widely accepted or standardized data preparation method. Indeed, it is quite difficult to create noiseless, clean data with minimal loss of information^18^. Based on our current knowledge of promoters, most sequences in a promoter appear to be required for recognition by RNAP^19^. However, with the exception of the −35 and −10 elements, other promoter sequences exhibit very low similarity. Furthermore, the distance between motifs and between motifs and TSSs are variable. For example, the distance between the −35 element and the −10 element can range from 14 to 20 nt, even in the same species. As a result, it is difficult to align the core promoters appropriately, which poses a challenge for data preparation. To overcome this obstacle, several researchers have directly translated the nucleotides in promoter sequences into digits, resulting in digital vectors that resemble the DNA sequences. Different approaches have been adopted to accommodate the variable distances between motifs, including initial sequence alignment^20^ and coupling SVM with a sequence alignment kernel to affine gaps in the input sequences^7^. In some studies, the DNA sequences were broken down into collections of oligomers tagged with information on their locations relative to TSSs^6,21^. Despite these efforts, it is still a challenge to properly deliver promoter features to machine-learning programs.

Here, we propose a new promoter analysis procedure called image-based promoter prediction (IBPP), which can extract features from training sequences simultaneously with information on their spatial relationship and then directly apply these features to predict new promoters. We introduced a template-like string termed an “image”, which covers the complete core promoter. The “image” can be viewed as certain features inserted into the strings of flexible gaps to preserve their spatial relationship. Like the PWM, IBPP conducts the prediction by calculating the similarity between a target sequence and the generated “image”. However, in contrast to PWM, IBPP generates this “image” automatically using training data. Because known machine-learning methods are not suitable for the construction of “images”, we incorporated an evolutionary approach for their generation. In other works, the evolutionary algorithm has been proven effective for generating complex features for promoter identification^14^. In the present work, the performance of IBPP was tested using *E. coli* σ70 promoters. The “images” trained through the evolutionary method were capable of distinguishing *E. coli* σ70 promoters from protein-coding sequences. Although this initial version of IBPP cannot yet effectively balance sensitivity and specificity for short sequences, it exhibited good accuracy in the analysis of promoters within long sequences.

## Methods

### Evolution-driven “image” generation

The generation of “images” begins with randomly generated seed-images. A seed-image has the same length as mature “images” of 81 bp. Seed-images contain 10 continuous random nucleotide characters, with other positions filled by ‘–’, indicating gaps. For each seed-image, the position of the continuous nucleotides is randomly determined.

**Figure 1:**
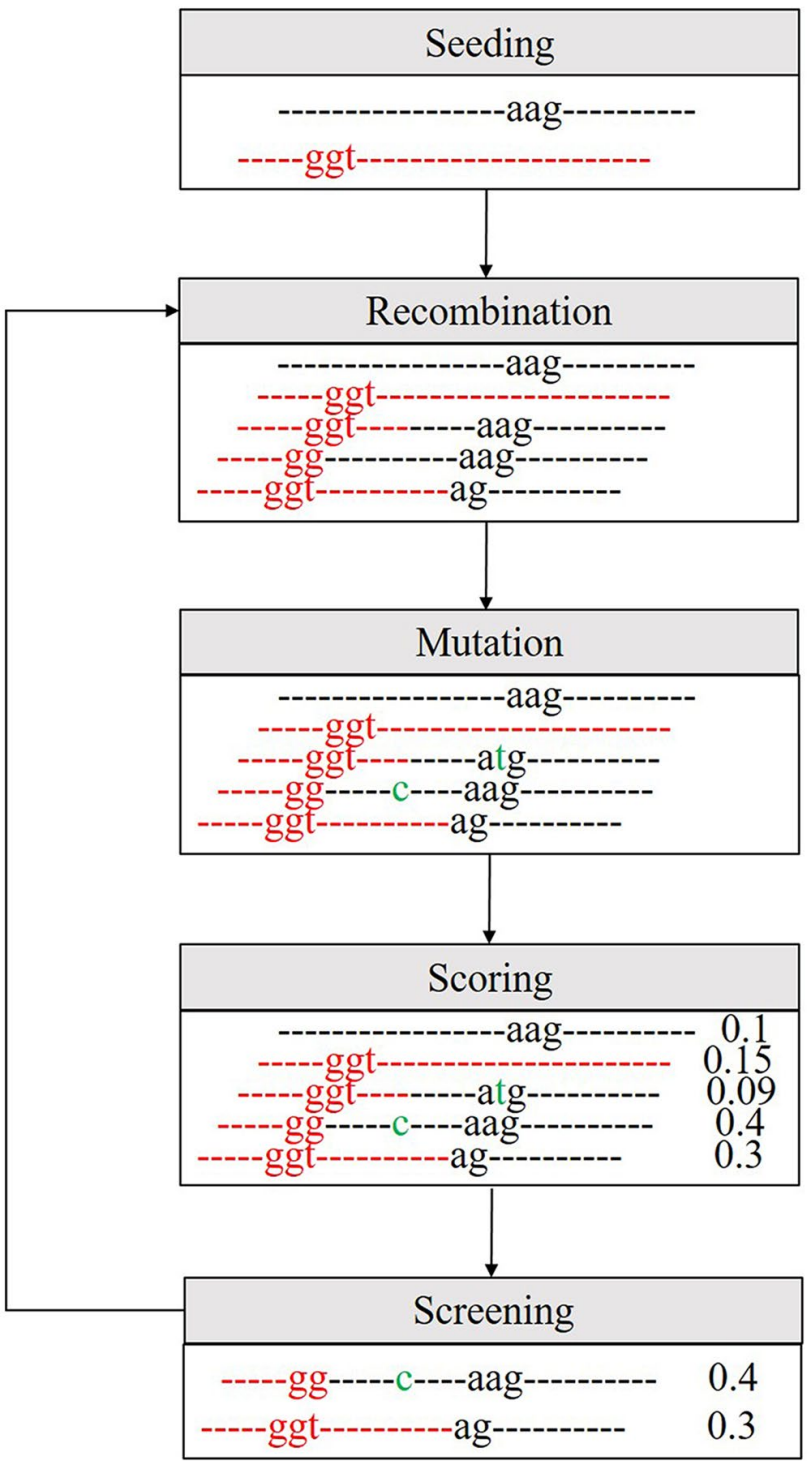
Workflow of the “image” generation process.

The evolution of “images” involves cycles of the following steps (Fig. 1):

1. Seeding: Initially, 20,000 independent seed-images were pooled into the “image” library. Because many “images” would be discarded after the selection step, 2,000 new seed-images were pooled into the “image” library whenever the number of “images” was smaller than 400.
2. Recombination: In each cycle, recombination occurred randomly for N (N = 2 × the size of the “image” library) times between any two “images” in the library. From two parental “images”, the recombination created a child “image” carrying the sequence from both parental images. The child “image” was the product of two crossovers at random positions between the parental images.
3. Mutation: In each cycle, 20% of the “images” in the “image” library developed random mutations. For these “images”, a single randomly selected character was randomly turned into a new character.
4. Scoring: Each “image” in the library was used to score all promoter and non-promoter sequences in the training sets. This score (D-score) was calculated as the difference between the mean scores of non-promoter and promoter sequences.
5. Screening: By comparing the D-scores, 90% of the “images” with lower D-scores were discarded. The remaining “images” in the library then entered the next cycle.

After a certain number of cycles, the “image” in the library with the highest D-score was then chosen for promoter analysis.

### Scoring

The score of each sequence was calculated by matching with an “image”. The “image” was a string of characters including {‘a’, ‘t’, ‘c’, ‘g’, ‘–’}. The four characters {‘a’, ‘t’, ‘c’, ‘g’} represent four different nucleotides, whereas ‘–’ represents gaps between nucleotides. To compare a sequence with an “image”, the sequence was first aligned with the “image”. Considering the variable gap lengths between promoter motifs, we decided to give the gaps a certain degree of flexibility. Like a spring, a string of gaps could be extended or compressed to a maximum of 20% when scoring. For example, if the “image” contained a series of gaps with a length of 10 nt, the program generated variations of this “image” with gap lengths of 8, 9, 11, and 12. Instead of comparing with one image, the sequence was aligned and scored with a series of gap-varied images. To align a sequence with an “image” or gap-varied image, the sequence was placed alongside the “image” and moved back and forth for 5 bp, resulting in 10 different alignments, and the highest score was recorded. The score was calculated using the following equation:

1 $$score={N}_{y}-0.4\times {N}_{x},$$

where *N*_*y*_ is the number of matched nucleotides, and *N*_*x*_ is the number of mismatches (gaps are not considered as mismatches).

### SVM analysis

IBPP was further coupled with SVM analysis (IBPP-SVM) to evaluate the effects of combining information from different “images” on the predictive performance. The vector corresponding to each sequence was comprised of scores calculated from several independently generated images; the dimension of vectors was determined by the number of “images” used. C-SVC coupled with the radical basis function kernel was used for solving the SVM problem, using LibSVM^22^ with gamma and C values of 0.1 and 1, respectively.

### Data sets

A total of 1,888 σ70 promoter sequences of *E. coli* K12 MG1655 (NC_000913) were retrieved from RegulonDB (version 9.0). Each promoter sequence spanned positions −60 to +20 relative to the TSS. The non-promoter sequences were randomly generated using the protein-coding sequences of *E. coli* K12 MG1655, which all had the same lengths as promoters. A collection of non-promoter sequences contained 10,000 sequences.

In the initial test of IBPP, 500 promoter and non-promoter sequences were randomly picked from each collection to build the training sets. For each test, the remaining sequences in both collections were used as the testing sets. The testing set for non-promoters had the same amount of sequences as the testing set for promoters.

To compare the performances of IBPP and IBPP-SVM on short sequences, 800 promoter sequences were randomly selected as a subcollection. To generate “images” for IBPP and IBPP-SVM, 500 sequences from the subcollection of promoters and 500 sequences from the collection of non-promoter sequences were randomly picked to build the training sets. For IBPP-SVM, the SVM algorithm was trained using the 800 promoter sequences and the first collection of non-promoter sequences. The remaining 1,088 TSSs and 1,088 sequences from the collection of non-promoter sequences were used as testing sets. To test the performance of IBPP and IBPP-SVM on long sequences, 2,000-nt-long sequences centered at the TSSs were retrieved from the genome of *E. coli* K12 MG1655.

For each test, the testing sets were confirmed to be free of contamination from the training sets.

### Analysis of long sequences

To analyze sequences with lengths of 2,000 nt, we used a sliding window approach with a step size of 1 and window size of 81 nt. For each position analyzed by IBPP, the score was recorded if it was above a threshold. However, with IBPP-SVM, the mean score calculated with several “images” was recorded for positions that tested positive. After the window slid through the sequence, scores were recorded for multiple positions. Positions with a distance of less than 50 nt were merged by ignoring the positions with smaller scores.

### Evaluation methods

For short sequences, the sensitivity, specificity, and F1 score were calculated as follows:

2 $$Sensitivity=\frac{TP}{TP+FN}$$

3 $$Specificity=\frac{TN}{TN+FP}$$

4 $${\rm{F}}1\,{\rm{score}}=\frac{2TP}{2TP+FP+FN}$$

where TP, TN, FP, and FN represent the numbers of true positives, true negatives, false positives, and false negatives, respectively.

For the long sequences, TP was defined as the number of sequences with a hit in the [−50, 50] range relative to TSSs. FP was defined as the number of hits in the range [−50, 50] divided by the length of this range. The sensitivity and specificity for long sequences were calculated as follows:

5 $${\rm{Sensitivity}}=\frac{TP}{Total\,number\,of\,sequ{\rm{e}}nces}$$

6 $${\rm{Specificity}}=\frac{TN}{TN+FP}$$

**Figure 2:**
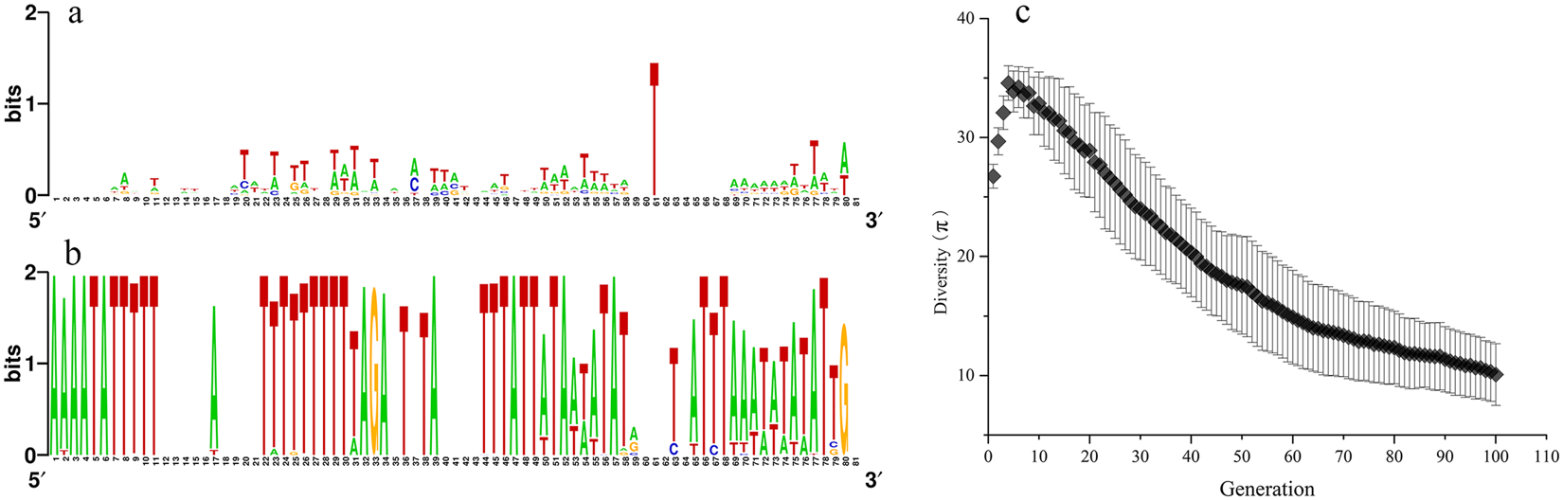
The generation of “images” from training sets. Analysis of 100 “images” with scores higher than others showed that the consensus was greater at the 100^th^ generation (**b**) than at the first generation (**a**). The diversity of the top 100 “images” in the library decreased during evolution (**c**). The results in (**c**) were obtained from 55 independent replications.

Nucleotide diversity π was calculated as described previously^23^. The sequence profile illustrated in Fig. 2 was generated by WebLogo^24^.

## Results and Discussion

### Generation of “images” by an evolutionary approach

The basis of IBPP is to generate an “image” from training sequences, which is then used to assess any target sequence and evaluate its similarity to the “image”. Starting from random seed-images, the final “images” were obtained using an evolutionary approach. The evolutionary method had a uniformization effect on the “images”, which was the foundation of this process. Although new seed-images were continually supplemented throughout the evolution process, we found that the diversity of the “images” showed a decreasing trend (Figs 2, S1). There was an increase in diversity in the first five generations caused by replacement of ‘–’ with nucleotide symbols. However, after the 60^th^ generation, the decrease in diversity slowed down (Fig. 2c). Although the diversity appeared to continue to decrease by the 100^th^ generation (Fig. 2c), we stopped the evolution at this point to test the predictive ability of the generated images.

Promoter and non-promoter sequences in the test sets were scored with the “image” generated at the 100^th^ generation. As shown in Table 1, the average score of promoter sequences was higher than that of non-promoter sequences (*p < *0.001). This showed that the “images” had the potential to distinguish promoters from non-promoters. With an appropriate threshold, sequences with scores above the threshold were predicted as promoters, whereas sequences with scores below the threshold were predicted as non-promoters. However, there was still some overlap between the scores of promoter and non-promoter sequences, indicating that the method could not simultaneously meet the requirements of both sensitivity and specificity.

**Table 1:** Comparison of scores of promoter and non-promoter sequences calculated using an “image”^a^.

	Promoter	Non-promoter
Score	10.9 ± 4.5	4.3 ± 3.5

^a^The average score of promoter sequences (±1 SD) was significantly higher that of non-promoter sequences (*p* < 0.001, independent t-test).

**Figure 3:**
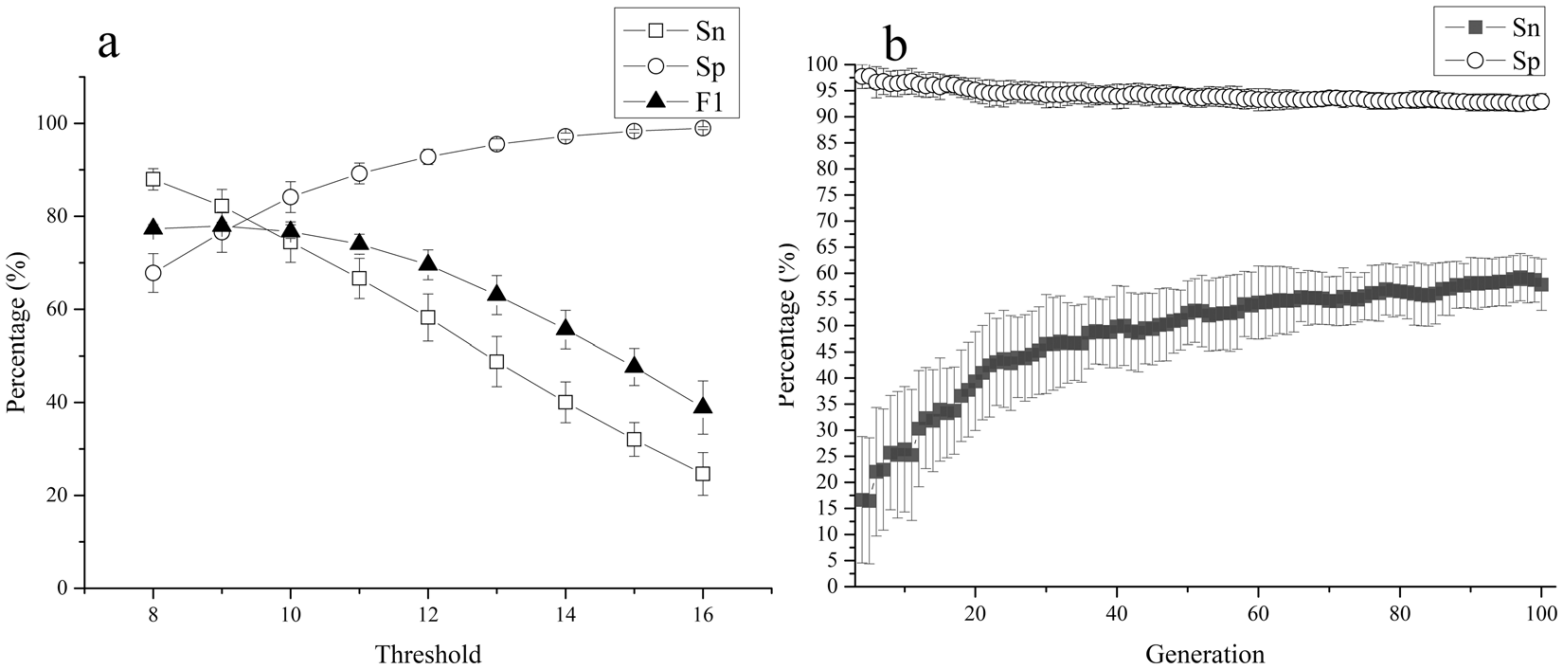
Promoter prediction ability of IBPP. The performance of IBPP on E. coli σ70 promoters was largely affected by the threshold (**a**). The predictive ability of the “image” increased along with evolution (**b**). The results are from 55 independent replications. Sn, sensitivity; Sp, specificity; FP, false-positive rate; TP, true-positive rate.

Indeed, the sensitivity was negatively correlated with the threshold (r = 0.999), whereas the specificity was positively correlated with the threshold (r = 0.999; Fig. 3a). When the threshold was below 9, IBPP acquired a sensitivity higher than 87%, but the specificity was reduced to below 70%. In our experiments, the highest F1 score was obtained with a threshold of 9 (F1 = 77.9%). However, in practice, a specificity below 90% can cause considerable issues. Therefore, a threshold of 12 is recommended, with a sensitivity and specificity of 58.2% ± 5.1% and 92.8% ± 1.6%, respectively. For promoters in other species, the optimum threshold may differ.

The predicted sensitivities of the “images” increased over the generations, showing a strong positive correlation in the fitted logarithmic regression model (r = 0.99; Fig. 3b). In the first 40 generations, the average TP increased from 0% to 47%, and then increased more gradually, ultimately reaching 58% in the 90^th^ generation. In contrast, the average FP did not change substantially over time, remaining near 5% for most generations. Considering that the predictive performance of the images exhibited only slight improvements after the 60^th^ generation and almost no change after the 90^th^ generation, 100 generations should be sufficient for promoters with a length of 81 bp. In our experiments, the preparation of images for shorter sequences required fewer generations.

The most well-known features of bacterial promoters are the −10 region (TATAAT) and −35 region (TTGACA). Because the “images” were trained from *E. coli* σ70 promoters, they should contain some features similar to the *E. coli* σ70 promoter. Although it was easy to detect the −10 region in all “images”, the −35 region was always more difficult to find. Moreover, the −10 region always appeared earlier than the −35 region. We assumed that this feature of earlier appearance may alter the appearance of other features. To evaluate the formation of different features, the algorithm was slightly modified. When a string of continuous nucleotide characters stably appeared, the region was fixed and was not used in the subsequent image-generation processes. Using this method, we investigated the effects of the mismatch penalty for image scoring on the formation of features. The results (Table S1) showed that lower penalty scores (0.4) caused the formation of longer continuous NTs. As the penalty score increased, the length of the continuous NTs was reduced. The –10 and –35 regions could be recognized as “features” with different lengths. Although “features” generated with a higher penalty score (0.75) seemed “clean”, the “images” generated with a lower penalty score yielded better results for promoter prediction (data not shown).

### Combining the SVM and evolutionary algorithm

“Images” generated in independent evolutionary processes showed certain diversity (data not shown), implying that different “images” may carry complementary information. Thus, although single “images” exhibited similar predictive ability, the combination of different “images” in one analysis may improve the predictive performance. To evaluate this combination effect, we employed SVM for promoter analysis using vectors consisting of values generated by different images.

**Figure 4:**
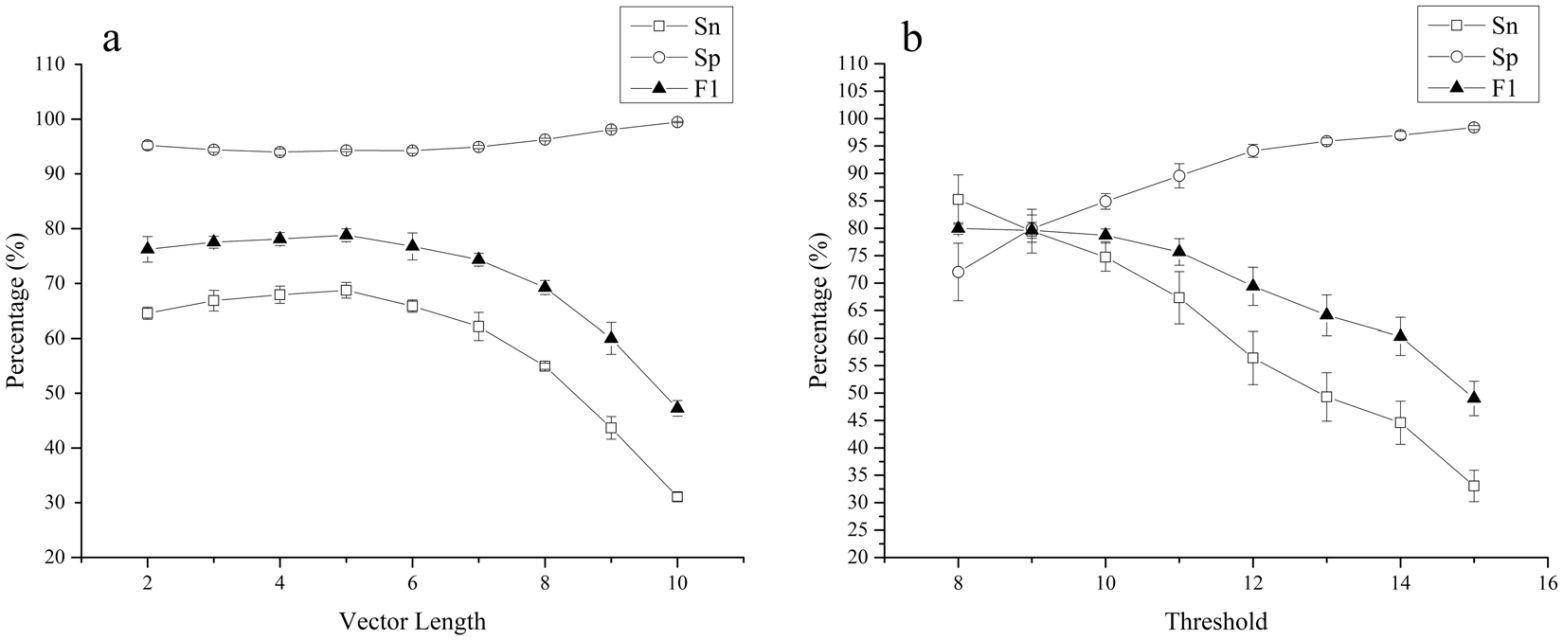
Promoter prediction ability of IBPP-SVM. The performance of IBPP-SVM was tested with vectors of different dimensions (**a**) and compared with IBPP (**b**). Both results were from three independent replications. Sn, sensitivity; Sp, specificity; FP, false-positive rate; TP, true-positive rate.

The sensitivity of IBPP-SVM for short sequences was largely affected by the dimension of the vectors (Fig. 4a). When the length of vectors was below 6, the sensitivity of IBPP-SVM increased slightly with increasing vector dimensions (e.g., 64.5% ± 1.1% for a 2-dimensional vector and 68.7% ± 1.4% for a 5-dimensional vector). However, the sensitivity decreased with higher dimensions, dropping to only 31.1% ± 0.8% for the 10-dimensional vector. In contrast, the specificity of IBPP-SVM for short sequences was not affected by vector length and was maintained at around 95% in all cases. The combination of different “images” by introducing SVM improved the performance for short sequences compared with IBPP. Under the same testing conditions, the best results obtained using IBPP-SVM (sensitivity = 68.7% ± 1.4%, specificity = 94.3% ± 0.2%) were significantly higher than those of IBPP with a threshold of 12 (sensitivity = 56.4% ± 4.9%, specificity = 94.1% ± 1.2%; Fig. 4b).

When the number of promoter sequences in the training dataset was fixed, a higher number of non-promoter sequences resulted in lower sensitivity but higher specificity (Fig. S2). The relationship between sensitivity and specificity was analyzed using a 5-dimensional vector, and the results demonstrated that the sensitivity decreased rapidly when the specificity was above 85%. Considering the overall performance, the best performance of IBPP-SVM was obtained with a sensitivity of 89.3% and specificity of 85.9%.

To evaluate the performances of IBPP and IBPP-SVM, the testing sets used to analyze the performance of NNPP2.2^11^ and BPROM^25^ were used for promoters and non-promoters. Using these same testing sets, NNPP2.2 obtained a sensitivity of 64.6% and specificity of 90.3%, whereas BPROM obtained a sensitivity of 95.7% and specificity of 98.9%. The F1 scores obtained by NNPP2.2 and BPROM were 74.1% and 97.3%, respectively. IBPP-SVM showed higher sensitivity and specificity than NNPP2.2, but the performance was still incomparable to that of BPROM. This result showed that IBPP-SVM could achieve an efficiency comparable to or even higher than some machine learning algorithms; however, further improvements are required to achieve a performance similar to that of BPROM.

The algorithm for IBPP does not require knowledge of features of promoters, such as the −10 region and −35 region of *E. coli* promoters. Unlike machine-learning programs, which rely on statistical data for the classification of promoters, the application of an “image” in promoter prediction is similar to that in the PWM approach. An “image” is comprised of strings of nucleotides and gaps between them; thus, the continuous nucleotides in an “image” resemble the features of bacterial promoter sequences, and the gaps resemble the spacing between features, restricting pseudofeatures to certain positions. Because such “images” cannot readily be constructed by machine-learning algorithms, such as SVM and ANN, we applied an evolutionary algorithm. Without any manual intervention, the “images” would self-improve with the help of the evolution process. Theoretically, this evolutionary system could be applied for drawing information from other types of sequences, such as ribosome-binding sites and coding sequences.

### Performance of long sequences

**Figure 5:**
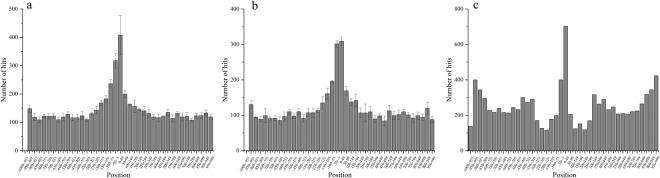
Analysis of long sequences using IBPP-SVM (**a**), IBPP (**b**), and BPROM (**c**) for sequences spanning the [–1000, 1000] region related to TSSs. The results of IBPP-SVM and IBPP were from three independent replications.

Next, we tested the ability of IBPP and IBPP-SVM to deal with longer sequences of 2,000 nt. For all tested methods, there were hits at positions close to or far from the TSSs simultaneously; however, the hits were more concentrated in the range of [−50, 50] relative to TSSs (Figs 5, S3). For long sequences, we adopted new definitions for sensitivity and specificity so that predictions with more hits in the [−50, 50] range exhibited higher sensitivity, while predictions with more hits outside of the [−50, 50] range exhibited lower specificity. Although BPROM outperformed IBPP-SVM for short sequences, the predictive ability of IBPP-SVM for long sequences was comparable to that of BPROM (Table 2). The best result of IBPP-SVM was obtained with 10-dimensional vectors, which was even better than that of BPROM considering both sensitivity and specificity. This is in large contrast to the analysis of short sequences, in which IBPP-SVM with 10 dimensional vectors showed very low sensitivity (31.08% ± 0.8%, Fig. 4). When the vector dimension was 5, IBPP-SVM showed the best performance for short sequences but did not exhibit good specificity for long sequences (Table 2). This comparison revealed that IBPP-SVM could achieve excellent performance with long sequences and that the performance was largely affected by the vector dimension.

**Table 2:** Performance of IBPP-SVM and IBPP for long sequences^a^.

	Sensitivity (%)	Specificity (%)
**IBPP-SVM**		
Dimension = 2	64.1 ± 3	64.6 ± 1.5
Dimension = 3	66.2 ± 2.7	65.5 ± 1
Dimension = 4	67.3 ± 2.1	65.2 ± 1
Dimension = 5	67.7 ± 1.2	64.7 ± 0.4
Dimension = 6	64.4 ± 2.3	64 ± 0.5
Dimension = 7	56.2 ± 0.5	63.5 ± 0.4
Dimension = 8	54.8 ± 0.4	64.3 ± 0.5
Dimension = 9	56.1 ± 1.7	67.1 ± 1.1
Dimension = 10	**65.9 ± 5.2**	**73 ± 2.1**
Dimension = 11	35 ± 17.3	77.7 ± 2.7
**IBPP**		
Threshold = 12	59.8 ± 1.4	69 ± 1.4
Threshold = 13	55.5 ± 1.7	73.6 ± 1.3
**BPROM**	57.5	69.5

^a^The values are averages from triplicate experiments ± SDs for IBPP-SVM and IBPP.

We then tested IBPP with thresholds of 12 and 13 on long sequences. Under both conditions, IBPP showed a sensitivity and specificity comparable to those of BPROM (Table 2). Although IBPP with a threshold of 12 had a slightly higher sensitivity, higher specificity was achieved with a threshold of 13. Because BPROM had such extraordinary performance for the analysis of short sequences, the performance of IBPP in the analysis of long sequences exceeded our expectations. This may be because BPROM was designed for intergenic sequences much shorter than 2 kb. In addition, as we only ran BPROM with the default set of parameters in this comparison, the performance of BPROM on long sequences may improve upon optimization of the parameters. Because bacterial genes are usually ~l kb in length, there may be more than one TSS in each fragment. Thus, some hits outside of the [−50, 50] range may be caused by other promoters in the fragments.

The sequences around promoters have the potential to bias the prediction algorithm. In this study, no obvious bias was detected for IBPP due to surrounding sequences. For example, when the threshold was 12, IBPP obtained TP and FP rates of 56% and 5.88% for short sequences, respectively, and a TP rate of 59% for long sequences. Therefore, the extended sequence length itself may not have a negative impact on the performance of IBPP. The reason for this difference could be related to the scoring system. Although the FP rate for short sequences was 5.88%, the scores of these FP sequences were lower than those of the TP sequences overall. When applied to long sequences, instead of being biased by these FP hits, such hits would be drawn toward the nearby TP hits. In addition, the combination of IBPP and SVM showed interesting results; similar to the performance on short sequences, the performance of IBPP-SVM for analysis of long sequences also exceeded that of IBPP. The effects of vector dimension on IBPP-SVM were largely different between long sequence and short sequence analyses. With a vector dimension of 10, the TP rate dropped to 31% for short sequences, accompanied by an FP rate of 2.3%, which was much lower than that with a vector dimension of 5. However, under the same conditions, IBPP-SVM with a vector length of 10 showed a TP rate of 65.6% for long sequences and a lower FP rate than that with a vector length of 5. Although we cannot currently explain this phenomenon, the results advanced us toward finding a resolution for further improvement of this prediction method on long sequences.

## Conclusion

In this study, we created a new promoter prediction algorithm called IBPP, which was combined with SVM (IBPP-SVM). IBPP used a dataset for a group of promoters characterized experimentally (e.g., with RNA-Seq) as training sequences to build a sequence template (“image”) through an evolutionary approach. The key to promoter prediction by IBPP and IBPP-SVM was comparison of sequences with the “image”. Both IBPP and IBPP-SVM could discriminate promoter and non-promoter sequences in long sequences (2,000 nt) or short sequences (81 nt).

Based on an algorithm that is fundamentally different from those presented in previous methods, IBPP showed good ability to identify *E. coli* σ70 promoters. IBPP analyzed sequences by matching them with an “image”, which imitated the organization of sequence features in a promoter. Similar to the PWM method, IBPP exhibited sequence features in an intuitive way. However, similar to machine-learning methods, the “image” was not designed manually but was instead trained from known sequences. When training with full-length promoter sequences, the “images” had more of a chance to include more sequence features than possible with the commonly used motifs in the PWM method. New “images” could also be trained using TSSs from other species, which may allow for cross-species promoter prediction. Although IBPP exhibited moderate performance on short DNA sequences, the combination of IBPP and SVM could further enhance its performance. IBPP showed good sensitivity and specificity when predicting promoters from long sequences. Although there is still substantial room for improvement, the evolutionary algorithm showed its ability to draw information from DNA sequences.

Nevertheless, there is still much room for improvement in the sensitivity of IBPP on short sequences. Since the creation of the “image” is largely affected by the training group of promoter and non-promoter sequences chosen, it may be possible to enhance performance by pre-characterizing and subgrouping the training set. The evolutionary algorithm used in this study was quite simple. In future studies, we are planning to integrate more parameters (e.g., GC content, stability) to the selective criteria of the evolutionary algorithm.

## Electronic supplementary material

Supplementary materials

## Data Availability

The datasets generated during and/or analyzed during the current study are available from the corresponding authors upon request.

## References

[1] Browning DF, Busby SJW (2016). Local and global regulation of transcription initiation in bacteria. Nat Rev Microbiol.

[2] Osbourn AE, Field B (2009). Operons. Cell Mol Life Sci.

[3] Staden R (1984). Computer methods to locate signals in nucleic-acid sequences. Nucleic Acids Res.

[4] Baldi P, Chauvin Y, Hunkapiller T, Mcclure MA (1994). Hidden Markov-models of biological primary sequence information. P Natl Acad Sci USA.

[5] Towsey M, Timms P, Hogan J, Mathews SA (2008). The cross-species prediction of bacterial promoters using a support vector machine. Comput Biol Chem.

[6] Gordon JJ, Towsey MW, Hogan JM, Mathews SA, Timms P (2006). Improved prediction of bacterial transcription start sites. Bioinformatics.

[7] Gordon L, Chervonenkis AY, Gammerman AJ, Shahmuradov IA, Solovyev VV (2003). Sequence alignment kernel for recognition of promoter regions. Bioinformatics.

[8] Silva SDE, Echeverrigaray S, Gerhardt GJL (2011). BacPP: Bacterial promoter prediction-A tool for accurate sigma-factor specific assignment in enterobacteria. J Theor Biol.

[9] Burden S, Lin YX, Zhang R (2005). Improving promoter prediction Improving promoter prediction for the NNPP2.2 algorithm: a case study using *Escherichia coli* DNA sequences. Bioinformatics.

[10] Pedersen AG, Baldi P, Brunak S, Chauvin Y (1996). Characterization of prokaryotic and eukaryotic promoters using hidden Markov models. Proceedings. International Conference on Intelligent Systems for Molecular Biology.

[11] Reese MG (2001). Application of a time-delay neural network to promoter annotation in the Drosophila melanogaster genome. Comput Chem.

[12] Bajic VB (2002). Dragon Promoter Finder: recognition of vertebrate RNA polymerase II promoters. Bioinformatics.

[13] Jiang B, Zhang MQ, Zhang XG (2007). OSCAR: One-class SVM for accurate recognition of cis-elements. Bioinformatics.

[14] Kamath, U., De Jong, K. A. & Shehu, A. An Evolutionary-based Approach for Feature Generation: Eukaryotic Promoter Recognition. *Ieee C Evol Computat*, 277–284 (2011).

[15] Narang V, Sung WK, Mittal A (2005). Computational modeling of oligonucleotide positional densities for human promoter prediction. Artif Intell Med.

[16] Mann, S., Li, J. Y. & Chen, Y. P. P. A pHMM-ANN based discriminative approach to promoter identification in prokaryote genomic contexts. *Nucleic Acids Res* **35**, 10.1093/nar/gkl1024 (2007). 10.1093/nar/gkl1024 PMC1802591 17170007

[17] Umarov, R. K. & Solovyev, V. V. Recognition of prokaryotic and eukaryotic promoters using convolutional deep learning neural networks. *Plos One* **12**, 10.1371/journal.pone.0171410 (2017). 10.1371/journal.pone.0171410 PMC5291440 28158264

[18] Zhang SC, Zhang CQ, Yang Q (2003). Data preparation for data mining. Appl Artif Intell.

[19] Ruff EF, Record MT, Artsimovitch I (2015). Initial Events in Bacterial Transcription Initiation. Biomolecules.

[20] Silva SDE, Gerhardt GJL, Echeverrigaray S (2011). Rules extraction from neural networks applied to the prediction and recognition of prokaryotic promoters. Genet Mol Biol.

[21] Lin H, Li QZ (2011). Eukaryotic and prokaryotic promoter prediction using hybrid approach. Theor Biosci.

[22] Chang, C. C. & Lin, C. J. LIBSVM: A Library for Support Vector Machines. *Acm T Intel Syst Tec* **2**, 10.1145/1961189.1961199 (2011).

[23] Nei M, Li WH (1979). Mathematical-Model for Studying Genetic-Variation In Terms Of Restriction Endonucleases. P Natl Acad Sci USA.

[24] Crooks GE, Hon G, Chandonia JM, Brenner SE (2004). WebLogo: A sequence logo generator. Genome Res.

[25] Solovyev, V. & Salamov, A. Automatic Annotation of Microbial Genomes and Metagenomic Sequences. *Metagenomics and its application in agriculture, biomedicine and environmental studies*, 61–78 (2011).

