# Supplementary material for: Image-based promoter prediction: a promoter prediction method based on evolutionarily generated patterns
Source: Sci Rep. 2018 Dec 6;8:17695. doi: 10.1038/s41598-018-36308-0 (PMC6283834; doi:10.1038/s41598-018-36308-0)
Supplement: Supplementary file 1 — Supplementary materials [file 41598_2018_36308_MOESM1_ESM.pdf]

Image-based promoter prediction: a promoter prediction method based on evolutionarily generated patterns

Sheng Wang[1], Xuesong Cheng[1], Yajun Li[1], Min Wu[1], and Yuhua Zhao[1,*]

[1]College of Life Sciences, Zhejiang University, Hangzhou, Zhejiang ZJ310058, China

* Address correspondence to Yuhua Zhao: yhzhao225@zju.edu.cn

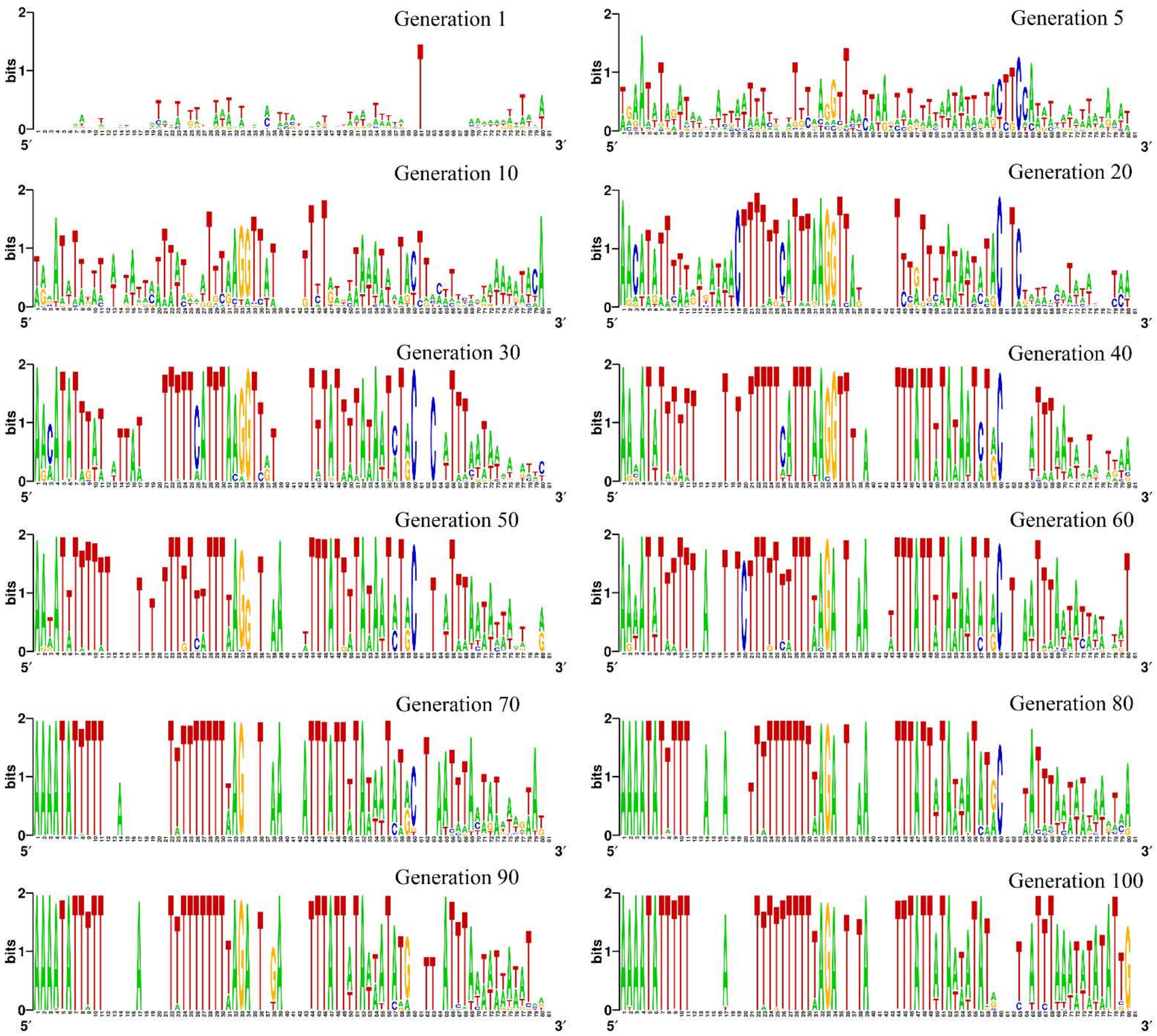

Figure S1. Change of diversity in the “image” library

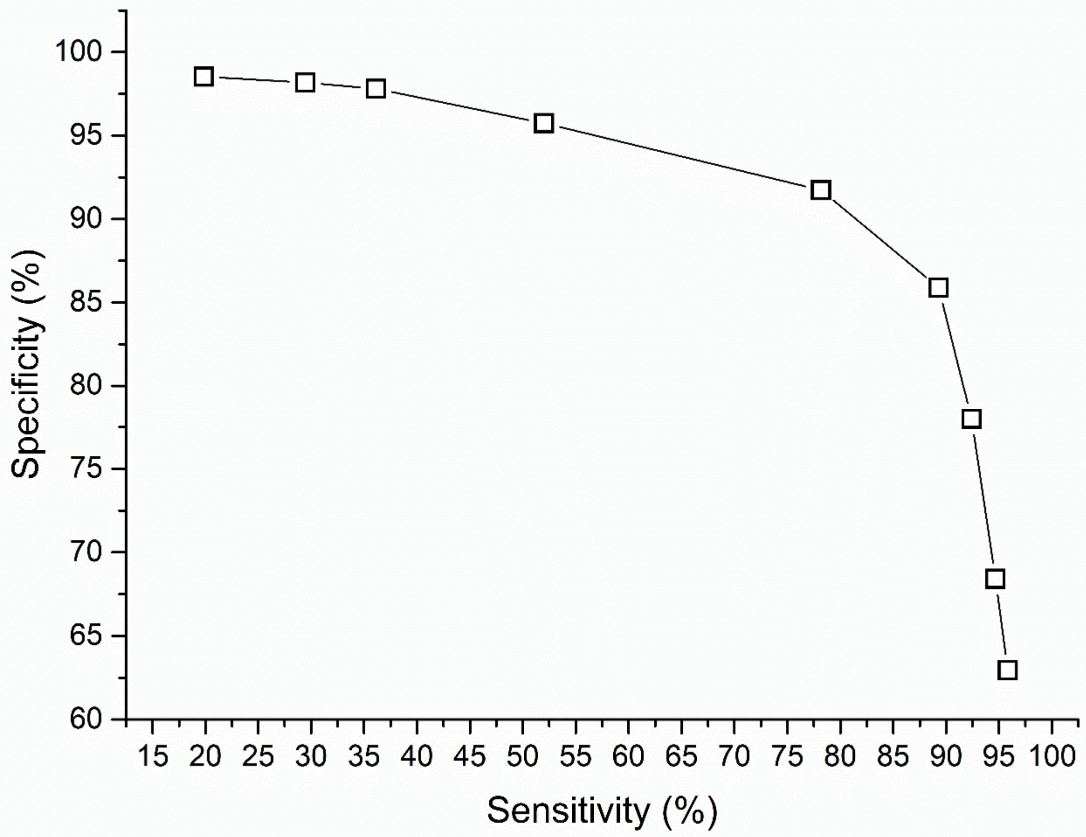

Figure S2. The sensitivity-specificity curve obtained using IBPP-SVM

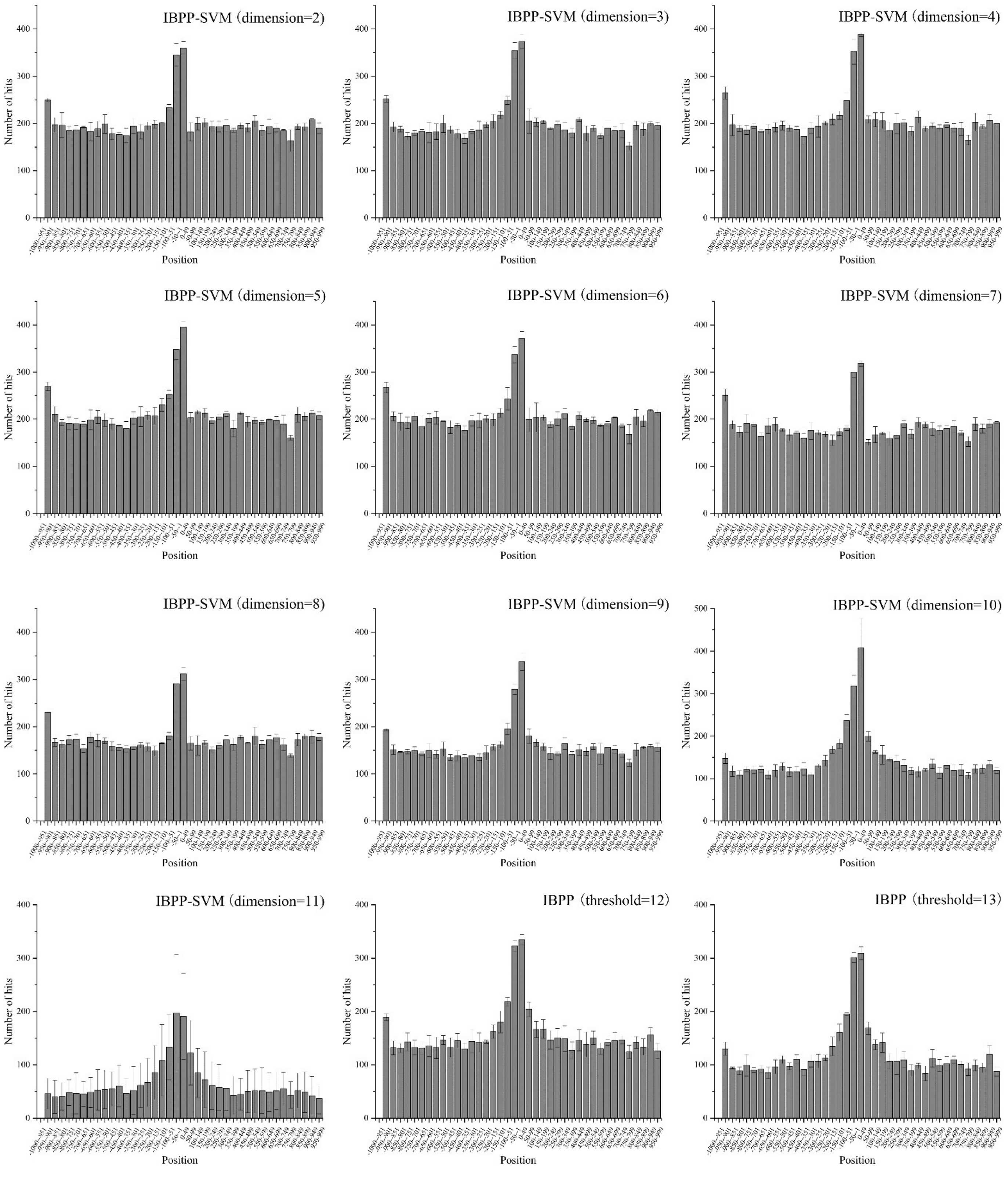

Figure S3. The analysis of long sequences using different methods

Table S1. The potential features generated by IBPP[a]

**Position[b]**	**Feature**
**Penalty = 0.75**	
-14	tataat
-36	ttga-a
2	ttaat
-26	att
-53	taat
**Penalty = 0.55**	
-36	ttgacattt
-22	ttaa-tgttataatat
-59	atattcatgaaattt
**Penalty = 0.4**	
-42	atcatttttgaaatt
2	atttaatgatgaaat
-21	aattgttataatataat

[a]Three mismatch penalties tested using the same dataset.

[b]The positions of the first NTs relative to the TSSs.
